# Anticariogenic Properties of *ent*-Pimarane Diterpenes Obtained by Microbial Transformation

**DOI:** 10.3390/molecules15128553

**Published:** 2010-11-25

**Authors:** Marcela E. Severiano, Marilia R. Simao, Thiago S. Porto, Carlos H. G. Martins, Rodrigo C. S. Veneziani, Niege A. J. C. Furtado, Nilton S. Arakawa, Suraia Said, Dioneia C. R. de Oliveira, Wilson R. Cunha, Luiz E. Gregorio, Sergio R. Ambrosio

**Affiliations:** 1 Nucleus of Research in Sciences and Technology, University of Franca, Franca, SP, Brazil; 2 Faculty of Pharmaceutical Sciences of Ribeirao Preto, University of Sao Paulo, Ribeirao Preto, SP, Brazil; 3 Institute of Research and Development, Vale do Paraiba University, Sao Jose dos Campos, SP, Brazil

**Keywords:** *Ent*-pimara-8(14),15-dien-19-oic acid, microbial transformation, diterpenes, anticariogenic properties, antimicrobial activity

## Abstract

In the present work, the anticariogenic activities of three pimarane-type diterpenes obtained by fungal biotransformation were investigated. Among these metabolites, *ent*-8(14),15-pimaradien-19-ol was the most active compound, displaying very promising MIC values (ranging from 1.5 to 4.0 μg mL^−1^) against the main microorganisms responsible for dental caries: *Streptococcus salivarius*, *S. sobrinus*, *S. mutans*, *S. mitis*, *S. sanguinis*, and *Lactobacillus casei*. Time kill assays performed with *ent*-8(14),15-pimaradien-19-ol against the primary causative agent *S. mutans* revealed that this compound only avoids growth of the inoculum in the first 12 h (bacteriostatic effect). However, its bactericidal effect is clearly noted thereafter (between 12 and 24 h). The curve profile obtained by combining *ent*-8(14),15-pimaradien-19-ol and chlorhexidine revealed a significant reduction in the time necessary for killing *S. mutans* compared with each of these two chemicals alone. However, no synergistic effect was observed using the same combination in the checkerboard assays against this microorganism. In conclusion, our results point out that *ent*-8(14),15-pimaradien-19-ol is an important metabolite in the search for new effective anticariogenic agents.

## 1. Introduction 

Dental plaque is defined as a biofilm consisting of cariogenic bacteria adhered to the tooth surface. It plays an important role in the development of dental caries [1,2], one of the main oral diseases affecting humankind [3,4]. *Streptococcus mutans* is considered one of the main cariogenic microorganisms, due to its ability to synthesize extracellular polysaccharides from sucrose, mainly water-insoluble glucan, and to initiate plaque formation [5]. Other aerobic bacteria such as *Enterococcus faecalis*, *Lactobacillus casei*, and other species of *Streptococcus* are also important in the late phase of dental biofilm formation [1].

Mechanical removal of the dental plaque is the most efficient procedure to prevent caries, but the majority of the population does not perform this removal efficiently [6]. Moreover, professional dental treatment is often very expensive and not readily accessible, especially in developing countries [3]. In this sense, the use of chemicals as a complementary measure is necessary and has demonstrated to be of great value in preventing the formation of tooth surface biofilms [7]. 

Nowadays, chlorhexidine (CHD) is considered the gold standard anticariogenic agent [6]; however, the regular use of oral care products containing this chemical are often associated with various adverse effects [3,8,9]. In addition, CHD is much less effective in reducing the levels of *Lactobacillus* species, which are strongly related to caries evolution [6]. All these problems, therefore, reinforce the great importance of finding new anticariogenic compounds.

According to several authors [10,11], drugs derived from natural products can serve not only as new drugs themselves, but also as lead compounds for chemical modifications that will furnish derivatives with better activity and pharmacokinetic properties, new mechanisms of action, and fewer adverse side effects [12,13]. Recently, it has been emphasized that these modifications can be performed by fungal biotransformation processes, which have presented various advantages over the use of conventional chemical reagents [14,15,16].

Several studies have emphasized the antimicrobial activity of diverse natural compounds against cariogenic pathogens, and some extracts and metabolites with potential anticariogenic properties have been described [1,6,9,17,18]. Recently, our research group has demonstrated that the diterpene *ent*-pimara-8(14),15-dien-19-oic acid (**1**, PA) is able to inhibit the growth of the main microorganisms responsible for dental caries with very promising minimal inhibitory concentration (MIC) values (ranging from 2.5 to 5.0 μg mL^−1^). In that work, we also pointed out that this metabolite could be used as a prototype for further modifications aiming to discover novel, safe and effective anti-caries agents [9]. 

In agreement with our early findings and as part of our ongoing efforts to explore the antimicrobial potential of diterpenes against the pathogens responsible for dental caries, our research group has decided to obtain derivatives of PA by fungal biotransformation processes and to investigate their anticariogenic properties. In the present work, we describe the preparation of such compounds as well as the effective antimicrobial activity displayed by the derivative *ent*-8(14),15-pimaradien-19-ol and its kinetic and synergistic properties against the main microorganism responsible for dental caries.

## 2. Results and Discussion 

**Figure 1:**
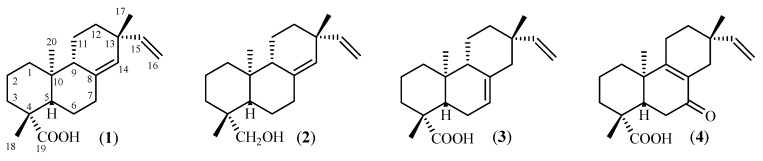
Chemical structures of PA (**1**) and its derivatives obtained by microbial transformation with *G. cingulata* (compound **2**) and *M. rouxii* (compounds **3** and **4**).

The incubation of PA (**1**) for 10 days with *Glomerella cingulata* afforded the main metabolite **2**, while its fermentation by *Mucor rouxii* (7 days) yielded the derivatives **3** and **4**. The chemical structures of the substrate (PA) and the diterpenes obtained by microbial transformation are presented in Figure 1. The spectral data of compounds **2 [19]**, **3** and **4** [20] are in agreement with those previously reported in the literature.

According to several authors [10,11], pharmacologically active metabolites derived from medicinal plants can serve not only as new drugs themselves but also as prototypes suitable for optimization through several semi-synthetic strategies. Still according to this approach, novel compounds with better pharmacological properties and/or fewer adverse side effects compared with their parent structure [14,15,16] can be achieved. Nowadays, microbial transformation has been considered an area of great interest for promoting selective modification in biologically active natural compounds, as it allows for the production of derivatives that are difficult to achieve by traditional organic synthesis.

Previous authors have investigated the microbial transformation of natural compounds by *G. cingulata* and pointed out that hydroxylation is the main reaction promoted by this fungus [21]. It has also been reported that this microorganism has the ability to promote the reduction of carboxylic acids to carbinols [22]. Analysis of the metabolites isolated in the present study showed that *G. cingulata* provided a 19.1% yield bioreduction in the carboxylic acid moiety of PA (**1**) to an alcohol group after a 10-day incubation. This finding is very interesting because chemical methodologies that promote carboxylic acid reduction are limited [23]. Furthermore, the yield of the biocatalytic reduction observed in this study was significant (19.1%), and future experiments to optimize the efficiency of this reaction can be performed. 

**Scheme 1:**
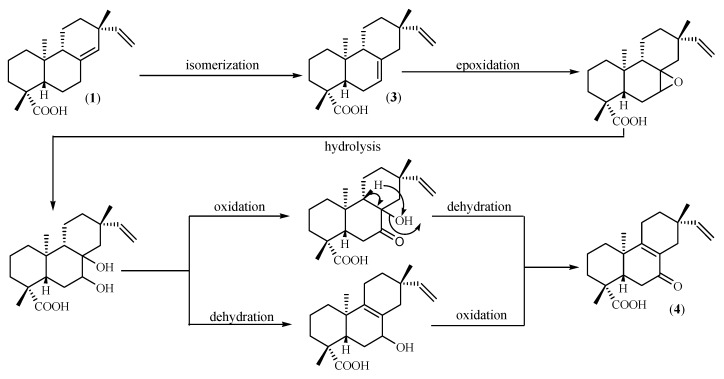
Suggested biogenetic pathway of compounds **3** and **4** according to Haridy *et al.* [24].

As for the other microbial biotrasformation agent, 7-day incubation of PA with *M. rouxii* led to two biotransformed diterpenes **3** and **4** (Figure 1). The structure of these metabolites indicates that isomerization of the endocyclic double bond and oxidation of the chemical structure of PA are the main reactions produced by *M. rouxii*. The suggested biogenetic pathway for **3** and **4** is presented in Scheme 1.

As mentioned before, our research group has concentrated its efforts on the evaluation of the antimicrobial potential of diterpenes, aiming to discover new natural anti-caries agents. In previous works [4,6,9], we have demonstrated that kaurenoic acid, copalic acid, and several pimarane type-diterpenes isolated from Brazilian plants can be used as prototypes for the production of new anti-infection agents against microorganisms responsible for this pathology. Among all the evaluated metabolites, PA (**1**, Figure 1) is one of the most active, displaying very promising MIC values [9] for the main pathogens responsible for dental caries. Moreover, we have observed that minor structure modifications drastically affect the referred antimicrobial activity [9]. In this sense, a multidisciplinary approach involving the discovery of novel active diterpenoids from natural products, combined with total or partial synthetic and biosynthetic methodologies should provide the best solution for exploring the anticariogenic potential of these metabolites. Additional antimicrobial studies to detail the kinetics, synergistic activities and mode of action of such compounds are also needed, in order to better understand new characteristics of this potential. 

In the present work, we have investigated the effect of three pimarane type-diterpenes, obtained by fungal biotransformation, against the main microorganisms responsible for human caries. The MIC and minimal bactericidal concentration (MBC) values displayed by these metabolites are shown in Table 1.

Among all the biotransformation products, compounds **2** and **3** are the most efficient, displaying very promising MIC values (lower than 10 μg mL^−1^) against all the investigated pathogens [25,26]. It is interesting to note that metabolite **2** is about two or three times more effective than its biosynthetic precursor **1** when considering the main cariogenic microorganism (*S. mutans*), thus denoting the importance of the fungal biotransformation as a source of new bioactive compounds. It is also noteworthy that these MIC and MBC values are much lower than some data recently reported in the literature for compounds belonging to other classes of natural products, such as triterpenoids [27], monoterpenes [28], phenolic compounds [8], and lignans [29]. 

**Figure 2:**
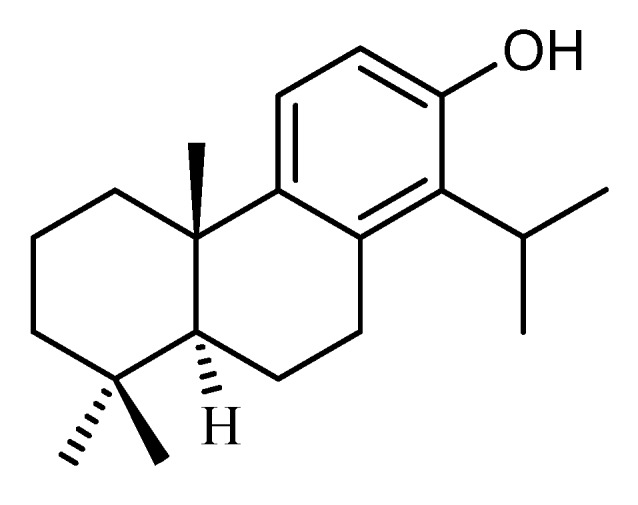
Chemical structure of totarol.

Considering other classes of diterpenes, searches in the scientific literature using PubMed and Scifinder databases and several keywords correlating diterpenes and caries diseases reveal that *ent*-pimaranes display one of the most promising antibacterial activities against oral pathogens [4,6,9,30,31,32,33,34,35]. A comparison of the MIC values against the main microorganism responsible for caries diseases obtained in the present study (*S. mutans*; Table 1) with those displayed by previously reported diterpenes [4,6,9,30,31,32,33,34,35] shows that compound **2** is only less active than totarol (Figure 2), which has a reported MIC value of 0.78 μg mL^−1^ (2.73 µM) [31]. This is a remarkable result, because very few natural products are known to inhibit the growth of oral pathogens [36]. All these results corroborate the great relevance of *ent*-pimarane type diterpenes, as well as that of the compound *ent*-8(14),15-pimaradien-19-ol (**2**) in the search for new effective anticariogenic agents. 

Although several diterpenes have been reported as being able to inhibit the growth of cariogenic bacteria with very promising MIC values [4,9], it is important to point out that further studies with these metabolites should be conducted, aiming at the future development of oral care products containing these natural compounds. In this context, the literature also describes additional experiments with the objective of investigating other features of the anticariogenic activity of diterpenes. For instance, time-kill curve experiments based on D´Arrigo *et al*. [37] and an investigation about a possible synergistic effect between the most efficient diterpene (compound **2**) obtained in the present study and CHD were performed against the primary causative agents responsible for dental caries.

**Figure 3:**
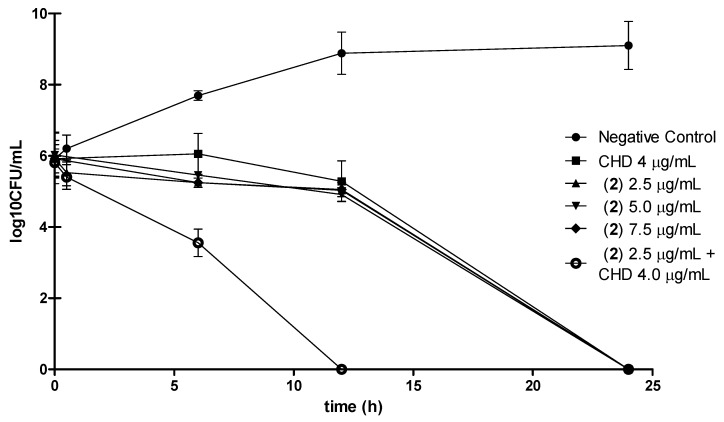
Time-kill curves for **2** in different concentrations and its association with CHD in their MBC values. Positive control: CHD.

Time-kill assay curves were constructed for *S. mutans* (5 × 10^5^ CFU mL^−1^) using three different concentrations of **2** (2.5, 5.0, and 7.5 µg mL^−1^ – one, two, and three-times its MBC value, respectively). A time-kill curve (Figure 3) was also constructed for **2** in association with CHD using their MBC values (2.5 and 4.0 µg mL^−1^, respectively), which allowed us to investigate the effect of the combination of these chemicals on the time necessary to achieve total elimination of the microorganism. 

The resulting time-kill curves presented in Figure 3 reveal that compound **2** alone only inhibits the growth of the inoculum (bacteriostatic effect) in the first 12 hours, while its bactericidal property is clearly noted thereafter (between 12 and 24 hours). Figure 3 also shows that this diterpene at 8.7 μM (2.5 µg mL^−1^) exhibits a time-kill curve profile that is very similar to those achieved with CHD at 6.9 μM (4.0 µg mL^−1^) (p < 0.05), thus indicating that CHD, the anticariogenic gold standard, is only slightly more potent than **2**. Moreover, in the same assay conditions, the curve profile displayed by this compound at 8.7 μM is also very close to that previously reported for the diterpene (-)-copalic acid (CA) at 23.1 μM [4]. These results allow us to conclude that **2** is about 2.5-fold more potent than CA, indicating that it is the most promising diterpene found by our research group so far. There were no statistical differences within the periods of time investigated for each concentration, leading to the conclusion that there were no dose-dependent responses in the experimental conditions (Figure 3). 

As for the synergistic antimicrobial test, the combinations of CHD with compound **2** did not exhibit any synergistic properties when the checkerboard methodology described by White *et al.* [38] was employed. According to this author, the obtained FIC index = 1.32 means “indifference”. Although this result clearly shows that the association of these chemicals did not improve their antibacterial activity, it is interesting to note that the combination of CHD with the diterpene **2** in their MBC values significantly reduced the time that was necessary to attain complete elimination of the main pathogen responsible for caries diseases, compared with the curve profiles obtained with these chemicals alone (Figure 3). 

Considering the relatively short period of time that oral care active ingredients remain in the oral cavity as well as the very promising MIC and MBC values obtained here, the time curve profile resulting from the combination of **2** and CHD also reveals that the association of active diterpenes with CHD brings new and significant perspectives to the development of novel oral care products capable of reducing the level of *S. mutans* more rapidly and efficiently. According to these results, it is possible to deduce that commercial botanical sources containing large amounts of diterpenes, such as the leaves of *Mikania glomerata* and *Salvia officinalis*, the oleoresin of *Copaifera* species, among others, should be investigated with a view to their application in the development of oral care products such as toothpastes and mouthwashes. 

Analysis of the chemical structure of several diterpenes and correlation between their structures and their ability to inhibit the growth of *S. mutans* [4,6,9,30,31,32,33,34,35] clearly shows that some structural features are essential for the antimicrobial activity displayed by these metabolites. Recently, Urzúa *et al.* [39] have suggested that diterpenes promote bacterial lysis and disruption of the cell membrane. According to these authors, the structural characteristics that promote the efficient antibacterial activity include a lipophilic structure, capable of insertion into the cell membrane, and one strategically positioned hydrogen-bond-donor group (HBD; hydrophilic group), which interacts with the phosphorylated groups on the membrane. In this study, it was also emphasized that a second HBD introduced in the lipophilic region or the absence of this hydrophilic group in the skeleton led to reduction in or suppression of the activity. 

A careful observation of the results depicted in Table 1 reveals that compounds **2** and **3,** which contain only one HBD at C-19, display much lower MIC values than those achieved with diterpene **4**, which contains two HBDs in its structure. This supports the structural hypothesis previously suggested by Urzúa *et al.* [39]. In addition, a comparison of the MIC values displayed by compounds **1** and **2** (PA) enables one to conclude that modifications to the HBD group change the antibacterial activity of these compounds. Moreover, MIC values can also be influenced by the position of the HBD group, as can be observed by the different MIC values obtained for compound **2** and the previously reported *ent*-8(14),15-pimaradien-3β-ol [9]. On the basis of the considerations that minor structural differences among the diterpenes significantly influence their antimicrobial activity, the results described in the present work can also be used to promote docking and QSAR studies, in association with data from other diterpenes reported in the literature. This kind of studies should certainly aid the comprehension of the structural features involved in the anticariogenic effect displayed by these natural compounds. 

## 3. Experimental 

### 3.1. General

Nuclear magnetic resonance (NMR) spectra were run on a Bruker DPX 400 spectrometer (400 MHz for ^1^H and 100 MHz for ^13^C). Samples were dissolved in CDCl_3_, and the spectra were calibrated with the solvent signals at δ 7.26 (^1^H) and δ 77.0 (^13^C). Biotransformation procedures were performed on a rotary shaker Cientec (CT-713). Vacuum liquid chromatography (VLC) [40] was carried out using silica gel 60H (Merck, art. 7736) in glass columns with 5-10 cm i.d. Medium pressure chromatography (flash chromatography) [41] was conducted with silica gel 60 (Merck, art. 9385) in a 450-25 mm glass column, using a flow rate at 5 mL/min. High performance liquid chromatography (HPLC) analyses were accomplished using a Shimadzu CBM-20A liquid chromatography controller, operating with the LCsolution software, equipped with a Shimadzu UV-DAD detector SPD-M20A and a Shimadzu ODS column (4.6 × 250 mm, 5 µm, 100 Å).

### 3.2. Isolation and identification of PA

Compound **1** (PA, 800.0 mg) was isolated from the dichloromethane root extract of Viguiera arenaria as previously described by Ambrosio et al. [42]. The spectral data (^1^H- and ^13^C-NMR) of PA are in agreement with those previously reported in the literature [19,43]. 

### 3.3. Microorganisms

The strains of Glomerella cingulata and Mucor rouxii used in the biotransformation processes were kindly assigned by Prof. Dr. Mônica T. Pupo and Prof. Dr. Suraia Said, respectively, both from the Faculty of Pharmaceutical Sciences of Ribeirão Preto, University of São Paulo. These microorganisms were maintained as a conidial suspension on silica gel (6-12 mesh, grade 40, desiccant activated), at 4 °C. 

### 3.4. Microbial transformation procedures

The microbial transformation experiments were performed using submerged shaken liquid culture by two-stage fermentation procedures. Initially, an inoculum of 1.5 × 10^5^ conidia mL^−1^ was aseptically added to two Erlenmeyer flasks (500 mL) containing 250 mL seed medium, as previously described by Jackson et al. [44]. Cultures were incubated with shaking on a shaker (120 rpm) at 30 °C. After 48 hours of incubation, the resulting mycelia were harvested, rinsed, and aseptically transferred to three Erlenmeyer flasks (1000 mL) containing 500 mL sterile medium composed by sucrose (3.0%), NaNO_3_ (0.2%), K_2_HPO_4_ (0.05%), MgSO_4_·7H_2_O (0.05%), KCl (0.05%), and FeSO_4_·7H_2_O (0.001%). After 24 hours, PA (400.0 mg) dissolved in dimethylsulfoxide (DMSO; 8 mL) was equally distributed among the three flasks and then incubated again. Incubation was stopped after 10 and 7 days, respectively, for Glomerella cingulata and Mucor rouxii. After that, the cultures were filtered, and their aqueous layer was extracted with ethyl acetate (5 × 500 mL; EtOAc), to furnish the extracts codified as GcE (G. cingulata extract; 723.0 mg) and MrE (M rouxii extract; 945.0 mg) after solvent evaporation. Culture (culture medium containing microorganisms incubated with DMSO only) and substrate (sterile medium containing the same amount of PA incubated under the experimental condition) controls were also performed for both strains.

### 3.5. Isolation of diterpenes 

Initially, GcE was chromatographed over silica gel using VLC with increasing amounts of EtOAc in n-hexane as eluent. This procedure furnished six fractions (300 mL each): GcE1 (58.0 mg; n-hexane), GcE2 (76.0 mg; 20% EtOAc), GcE3 (98.4 mg; 40% EtOAc), GcE4 (123.7 mg; 60% EtOAc), GcE5 (115.6 mg; 80% EtOAc), and GcE6 (213.0 mg; EtOAc). Fractions GcE3 and GcE4 were combined on the basis of their similar thin layer chromatography (TLC) profiles. Compound **2** [ent-8(14),15-pimaradien-19-ol; 73.0 mg] was obtained from this new combined fraction through flash chromatography using isocratic n-hexane-EtOAc (7:3) as mobile phase, and a flow rate of 5 mL min^−1^. ^1^H-NMR spectra analysis of fractions GcE1, GcE2, GcE5, and GcE6 did not reveal any characteristic signals of ent-pimarane type diterpenes. 

The fractionation of MrE was performed as described above, and the VLC chromatography gave additional ten fractions (MrE1-MrE10). On the basis of its TLC profile, fraction MrE3 (96.0 mg) was further fractionated by flash chromatography (n-hexane-EtOAc 8:2). TLC analysis of the sub-fraction MrE3.3 (55.0 mg) showed a main spot, which was later purified by classic chromatography and isocratic n-hexane/ EtOAc-CHCl_3_ (7:1:2) mobile phase, to give compound **3** (ent-pimara-7,15-dien-19-oic acid; 11.3 mg). A sample of MrE4 (49.4 mg) was analyzed by reversed phase HPLC using an analytical Shimadzu ODS column (MeCN-H_2_O 9:1 + 0.1% H_3_PO_4_; flow rate 1 mL min^−1^; UV detection at 210 nm), and compound **3** was also identified as the main constituent of this fraction. Fractions MrE5 and MrE6 were combined on the basis of their similar HPLC profile, and this new combined fraction (MrE5; 179.8 mg) was chromatographed using classic chromatography (n-hexane-EtOAc 7:3 + 1% acetic acid). Approximately 20.0 mg of the sub-fraction MrE5.2 (84.3 mg) was further purified by reversed phase HPLC using an analytical column. After repeated injections (50 μL; MeOH-H_2_O 9:1; flow rate 1 mL min^−1^; UV detection 210 nm) of samples containing 0.5 mg each, compound **4** (8.5 mg; 7-keto ent-pimara-8,15-dien-19- oic acid) was purified.

### 3.6. Bacterial strains and antimicrobial testing

The lowest concentration of the compound capable of inhibiting microorganism growth (MIC, (MIC, minimal inhibitory concentration) and the lowest concentration of the compound at which 99.99% or more of the initial inoculum was killed (MBC, minimal bactericidal concentration) were determined in triplicate by using the microdilution broth method in 96-well microplates [9]. The strains of the microorganisms used in this work were obtained from the American Type Culture Collection (ATCC): *Enterococcus faecalis* (ATCC 4082), *Streptococcus salivarius* (ATCC 25975), *Streptococcus sobrinus* (ATCC 33478), *Streptococcus mutans* (ATCC 25275), *Streptococcus mitis* (ATCC 49456), *Streptococcus sanguinis* (ATCC 10556), and *Lactobacillus casei* (ATCC 11578).

Samples were dissolved in dimethyl sulfoxide (DMSO; Synth) at 1 mg mL^−1^, followed by dilution in tryptic soy broth (Difco), and concentrations ranging from 200.0 to 1.0 μg mL^−1^ were achieved. The final DMSO content was 5% (v/v), and this solution was used as negative control. The inoculum was adjusted for each organism, to yield a cell concentration of 5 × 10^5^ colony forming units (CFU) per mL, according to guidelines of the Clinical Laboratory Standards Institute. One inoculated well was included, to allow control of the adequacy of the broth for organism growth. One non-inoculated well, free of antimicrobial agent, was also employed, to ensure medium sterility. Chlorhexidine dihydrochloride (CHD) was used as positive control. The microplates (96 - wells) were sealed with plastic film and incubated at 37 °C for 24 h. After that, resazurin (30 μL) in aqueous solution (0.02%) was added to the microplates, to indicate microorganism viability [9]. Before addition of resazurin and in order to determine MBC, an aliquot of the inoculum was aseptically removed from each well presenting no apparent growth and then plated onto tryptic soy agar supplemented with 5% sheep blood. The plates were incubated as previously described. 

### 3.7. Time-kill curves

Time-kill assays were performed in triplicate based on D’Arrigo *et al.* [37] against *Streptococcus mutans*, which is considered one of the primary causative agents of dental caries [1]. Compound **2** was chosen for the time-kill curve assays because it displayed the highest antimicrobial activity.

Tubes containing compound **2** at final concentrations of 2.5, 5.0, and 7.5 µg mL^−1^ (respectively one, two, and three-times the MBC of **2** for *S. mutans*) were inoculated with the tested microorganism, resulting in a start bacterial density of 5 × 10^5^ CFU mL^−1^, and then incubated at 37 °C. Samples were removed for determination of viable strains at 0, 0.5, 6, 12, 18, and 24 hours after incubation, followed by dilution, when necessary, in sterile fresh medium. The diluted samples (50 µL) were spread onto tryptic soy agar plate supplemented with 5% sheep blood, incubated at 37 °C, and counted after 48 h. Time-kill curves were constructed by plotting log_10_ CFU mL^−1^ versus time. The assays were performed in triplicate for each concentration and also for the positive (CHD, 4 µg mL^−1^) and negative controls (suspension of *S. mutans* without added compound **2**). CHD was used at its MBC value (4 µg mL^−1^).

In a second set of experiments, which aimed to investigate the effect of the combination of **2** with CHD on the time necessary to promote complete elimination of *S. mutans*, a time-kill curve was performed with these chemicals. CHD and **2** were used at their MBC values, and the same protocol described above was employed in this assay.

### 3.8. Synergistic antimicrobial activity

Checkerboard assays were accomplished according to the protocol previously described by White *et al.* [38]. The objective was to investigate the *in vitro* antimicrobial efficacy of the combination of CHD with **2**. The synergy tests were evaluated in triplicate, and concentrations of each compound (1/32 to 3 times their MIC values) were combined in standard MIC format against 5 × 10^5^ CFU mL^−1^ of *S. mutans*. To evaluate the synergistic effect between these chemicals, the fractional inhibitory concentration (FIC) index values were calculated on the basis of the equation previously established in the literature [38]. FIC index values were analyzed as follows: FIC index values ≤ 0.5, synergism; FIC index values > 4, antagonism. Indifference was defined as 0.5 < FIC index ≤ 4 [38].

## 4. Conclusions 

Our results allow us to conclude that *G. cingulata* and *M. rouxii* were able to promote the biotransformation of PA, leading to the formation of three metabolites. Considering all the aspects of the anticariogenic activity reported in this work, *ent*-8(14),15-pimaradien-19-ol was shown to be two or three times more active than its biosynthetic precursor when considering the main cariogenic microorganism (*S. mutans*). This reinforces the use of microbial biotransformation as an important strategy to promote modification on natural metabolites in the search for new bioactive compounds.

## Figures and Tables

**Table 1:** ***In vitro*** antibacterial activity (Minimum Inhibitory Concentration and Minimum Bactericidal Concentration, μg mL^−1^) of the biotransformed diterpenes against oral pathogens.

Compound	Microorganism					
	*L. casei*	*S. mitis*	*S. mutans*	*S. sanguinis*	*S. sobrinus*	*S. salivarius*
1*	3.0	4.0	4.5	2.5	4.0	5.0
**2**	2.5 (5.0)	1.5 (3.0)	1.5 (2.5)	3.5 (7.0)	4.0 (7.0)	3.5 (7.0)
**3**	5.0 (12.0)	4.0 (7.0)	6.0 (9.0)	8.0 (15.0)	7.5 (7.5)	5.0 (10.0)
**4**	160.0 (**)	200.0 (**)	200.0 (**)	120.0 (200.0)	200.0 (**)	180.0 (**)
**PC**	0.9	3.6	0.9	3.6	0.9	0.9

* previously reported MIC values [9]; ** MBC values higher than 200 μg mL^−1^; Positive Control (PC) – chlorhexidine dihydrochloride; Negative control (5% DMSO solution) did not affect the growth of the microorganisms.
